# Sterility and bioactivity evaluation of two types of bone graft substitutes after removing the original packaging

**DOI:** 10.34172/japid.2023.007

**Published:** 2023-04-11

**Authors:** Mehdi Ekhlasmand kermani, Aida Kheiri, Reza Amid, Maryam Torshabi, Behzad Houshmand, Sepideh Parsayan

**Affiliations:** ^1^Department of Periodontics, School of Dentistry, Kerman University of Medical Sciences, Kerman, Iran; ^2^Department of Periodontics, School of Dentistry, Shahid Beheshti University of Medical Sciences, Tehran, Iran; ^3^Dental Research Center, Research Institute of Dental Sciences, School of Dentistry, Shahid Beheshti University of Medical Sciences, Tehran, Iran; ^4^Department of Dental Biomaterials, School of Dentistry, Shahid Beheshti University of Medical Sciences, Tehran, Iran; ^5^Dental Student, School of Dentistry, Shahid Beheshti University of Medical Sciences, Tehran, Iran

**Keywords:** Allograft, Bioactivity, Bone graft, Sterility, Xenograft

## Abstract

**Background.:**

Xenograft and allograft bone substitutes are widely used to replace the missing bone in defects. Since removing the packaging of these grafts can nullify their sterilization, this study aimed to evaluate the sterility and bioactivity changes of an allograft and a xenograft following uncapping/recap.

**Methods.:**

Two types of commercial allograft and xenograft vials were unpacked and further exposed to operating room air, where implant surgery was performed for one second, ten minutes, and one hour. After three repetitions, samples were analyzed using microbiological tests and scanning electron microscopy (SEM) with energy dispersive x-ray analysis (EDX) for sterility and bioactivity evaluation.

**Results.:**

None of the bone graft samples showed microbial growth or bioactivity-negative changes after seven days of unpacking the vials.

**Conclusion.:**

Despite the positive results of this study, future studies and more analysis considering influential factors are required. Also, disinfection and air exchange must still be observed during biomaterial application and bone grafting procedures.

## Introduction

 Bone defects following cancer, trauma, periodontal disease, and tooth extraction have challenged clinicians.^1,2^ Bone grafting has been introduced as A beneficial treatment approach to replace the missing bone in the defects that cannot be healed spontaneously.^3,4^ Although autogenous bone grafts are still considered the gold standard, some shortcomings limit their use.^4-6^ Allografts are the second substitutes that have overcome some drawbacks but still have disadvantages, including limited supply and inappropriate sterilization methods.^7,8^ Xenograft materials are prepared following the removal of antigens and organic matter from species other than humans.^8^ Although these bone grafts are biocompatible and osteoconductive and have similar porous microstructure to human bone, processing methods, time-consuming procedures, and high prices can be named as their disadvantages.^7,9,10^ Synthetic materials represent the last alternative, mainly composed of biphasic calcium phosphate, calcium sulfate, and bioactive glass. These materials are not only safe but also prepared at a low price. Still, their unclear clinical efficiency and lack of osteoconductivity compared to the gold standard graft materials can be named as their drawbacks.^10,11^

 One of the principles in preparing allografts and xenografts is to remove organic materials, antigens, bacteria, and viruses.^11-13^ Various techniques, including gamma irradiation, ethylene oxide, chemical processing, and antibiotic soaks, have been used to sterilize allografts and xenografts and reduce the risk of disease transmission and subsequent cross-infection.^13,14^

 A sterility test is one of the examinations needed to assess any contamination in sterilized bone grafts.^15^ Moreover, scanning electron microscopy (SEM) and energy dispersive x-ray spectroscopy (EDX) are two other possible tests for the physicochemical and bioactivity evaluation of biomaterials. SEM imaging technique provides information regarding the morphology of granules, while EDX analysis shares information on the chemical elements present in the biomaterials and surrounding tissues, qualitatively and quantitatively. This method can also calculate the calcium-to-phosphate (Ca/P) ratio, which is a good bone mineralization indicator.^16,17^

 Bone grafts are marketed in pre-weighed vials, and according to the manufacturer’s instructions, each vial should be used for only one patient. In cases where there are some residual graft materials, they should be discarded. The risk of contamination and cross-infection increases since the bone grafts are exposed to air after opening the vial. Hence, the present study aimed to evaluate the sterility and bioactivity of two commercial bone graft substitutes (allograft and xenograft) after being removed from the manufacturer’s original packaging at room atmosphere using microbiological and SEM/EDX analyses.

## Methods

###  Experimental design’s ethics

 The detailed study design was analyzed and approved by the Ethics Committee of the Dental School, Shahid Beheshti University of Medical Sciences, with the following ID: IR.SBMU.DRC.REC.1398.208.

###  Sterility assay

**Figure 1:**
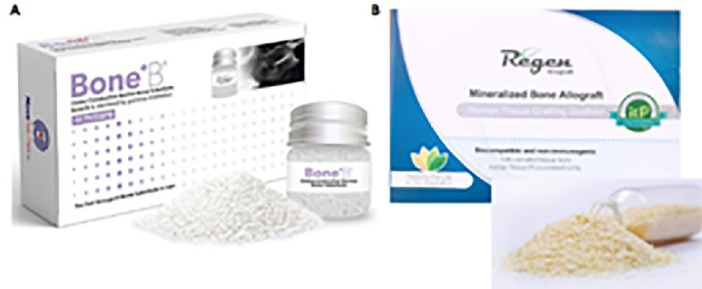
Xenograft (A) and allograft (B) types used in this study

 This study was performed in the operating room of the Periodontology Department of Shahid Beheshti (SBMU) Dental School during implant surgery. Bone grafts used in this study were mineralized bone allograft (ITP Co., Iran) and Bone ^+^ B^®^ bovine-sourced xenograft (NovaTebPars, Iran) in the particle size range of 1000‒2000 µm (Figure 1). The vials were removed from their original package and placed in three different operating room locations to investigate whether microbial loads were different in these locations or not. In a 36-m^2^ room, the selected locations were within the same radius of 1 meter from the dental unit where the surgery was performed. The exposure times to the room atmosphere were 1 s, 10 min, and 1 h (three replicates). In addition to test groups, brain heart infusion (BHI) (Conda, Spain) agar plates were placed in the same locations and conditions (opening the lid for 1 second, 10 minutes, and 1 hour) as control groups. All efforts were made to keep other influential factors, such as infection control, surgery procedures, and hand/instrument contamination, unchanged during our investigation. At the time of the study, implant surgery was performed by a single surgeon and assistant trained in the standard infection control protocol. The instrument used in the operating room was sterilized, and the surgical handpiece was used at the same speed of 1000 revolutions per minute (rpm).

 After the above-mentioned exposure times, recap vials and relid plates were transferred aseptically to the oral microbiology laboratory in aseptic conditions. The plates were incubated at 37 °C (95% humidity) and monitored for colony formation on semisolid culture media after 24 and 48 hours. Immediately and seven days after materials’ exposure to air, 50 mg of each bone graft sample was added to 4 mL of BHI broth, incubated at 37 °C, and monitored for turbidity of liquid culture media after 24 and 48 hours. For more precise evaluation, 100 µL of each sample was cultured on a BHI agar plate, incubated for 24 hours at 37 °C (three replicates), and monitored for colony formation. All microorganisms except the obligate anaerobes were studied.

###  In vitro bioactivity assay

 To investigate the bioactivity and physicochemical changes of bone grafts, seven days after unpacking the vials, 21 mg of each sample powder was added to 14 mL of simulated body fluid (SBF) (1.5 mg/mL).^18^

 The test tubes containing test bone grafts immersed in SBF were placed in a shaking incubator (120 rpm) at 37 °C for seven days. The SBF solution was removed and refreshed every two days. One and seven days after incubation, the samples were air-dried and analyzed with SEM/EDX.

 All the pre-defined procedures and evaluations were repeated three times.

###  Statistical analysis 

 The quantitative data were analyzed using GraphPad Prism software. T-test and one-way ANOVA were used to compare the groups concerning each variable. *P*< 0.05 was considered statistically significant.

## Results

###  Microbial evaluation of the room atmosphere

**Figure 2:**
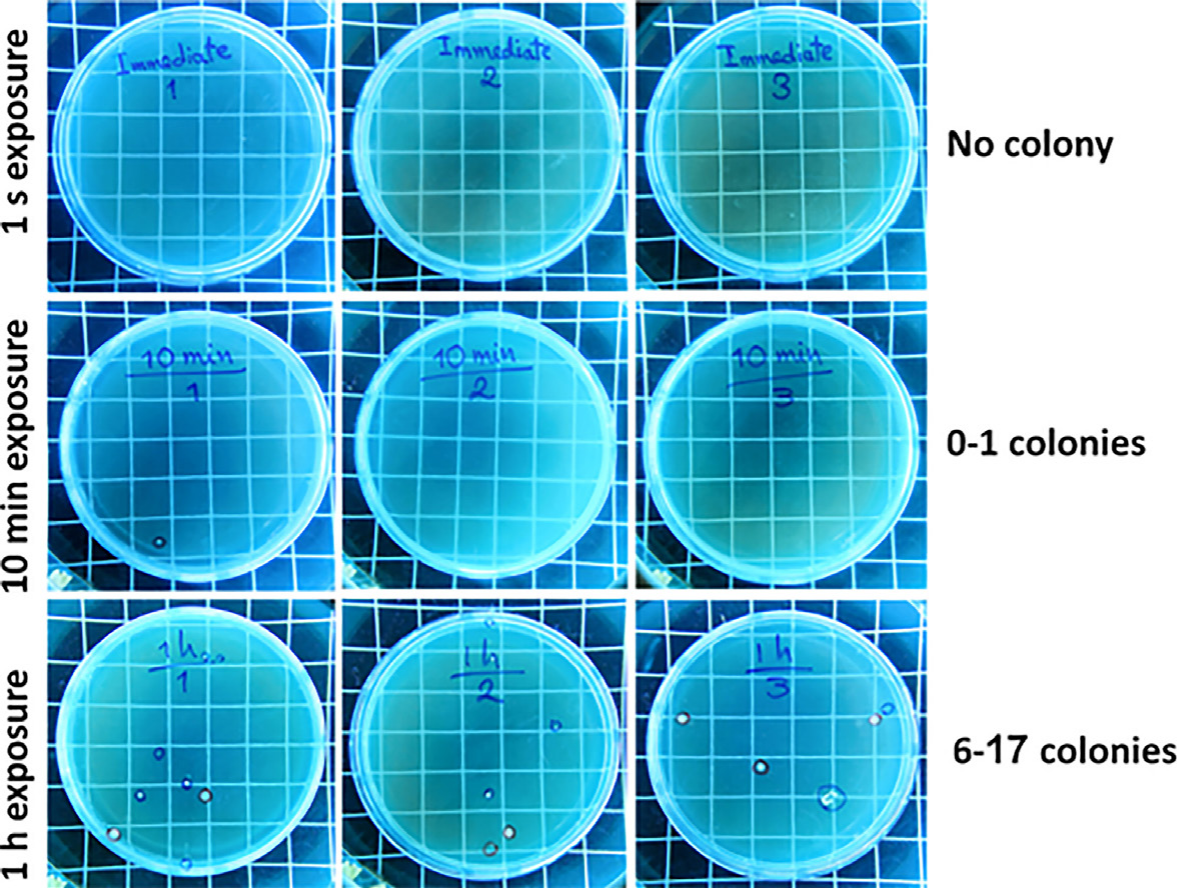
Colony formation evaluation on BHI agar plates (unlid/relid for 1 s, 10 min, and 1 h in the operating room) (n = 3) after 48 hours of incubation at 37 °C

 Control BHI agar plates (unlid for 1 second, 10 minutes, and 1 hour) were assessed after 24 and 48 h of incubation at 37 °C. No colony formation was observed on 1s-unlid plates after 24 and 48 hours of incubation. However, the plates exposed for 10 minutes showed colony formation (0 < colony < 1) after 24 and 48 hours of evaluation. The plates with 1 hour of air exposure showed 2‒3 and 6‒17 colonies after 24 and 48 hours, respectively (Figure 2).

###  Microbial evaluation of bone grafts

**Figure 3:**
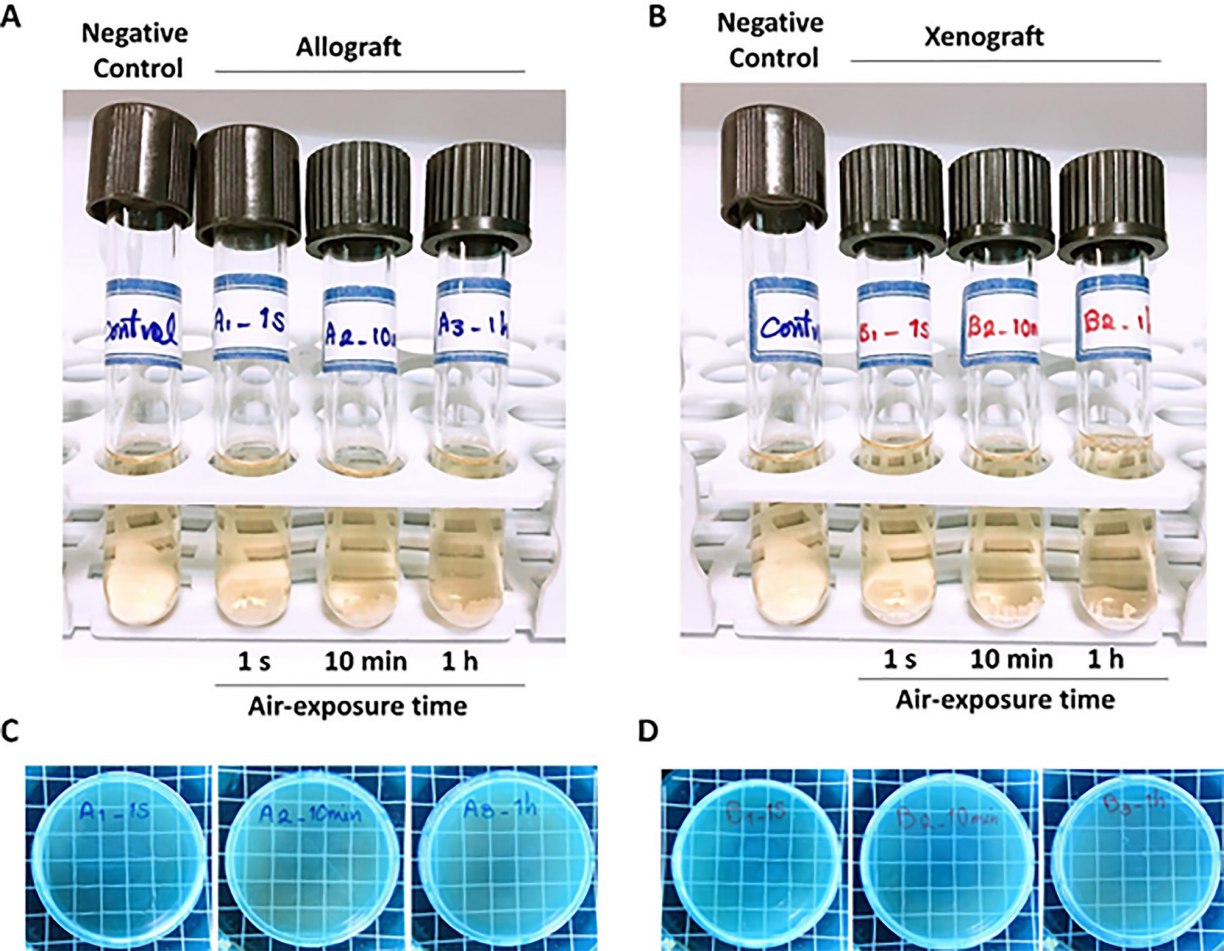
Visual turbidity (A and B) and colony formation (C and D) evaluation of the allograft (A and C) and xenograft (B and D) samples seven days after uncapping/recapping (1 s, 10 min, and 1 h in the operating room; n = 3)

**Figure 4:**
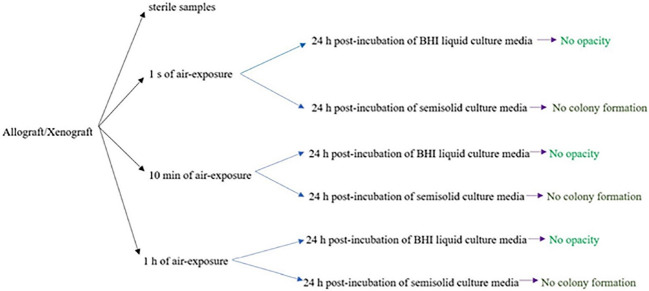
A summary of 24 hours post-incubation of samples in BHI liquid culture media and semisolid culture media

 Following seven days of uncapping/recapping (1 second, 10 minutes, and 1 hour of air exposure) and 24 hours of incubation of immersed test bone graft samples in BHI broth, no turbidity was observed (Figures 3A and 3B). In addition, no colony formation was observed on the related BHI agar plates, which complied with the visual assessment findings (Figures 3C and 3D). These findings also demonstrated no difference in microbial load in locations where the plates and samples were placed during surgery. Figure 4 shows a summary of the 24-hour post-incubation period of samples in BHI liquid culture media and semisolid culture media.

###  Bioactivity evaluation of bone grafts

**Figure 5:**
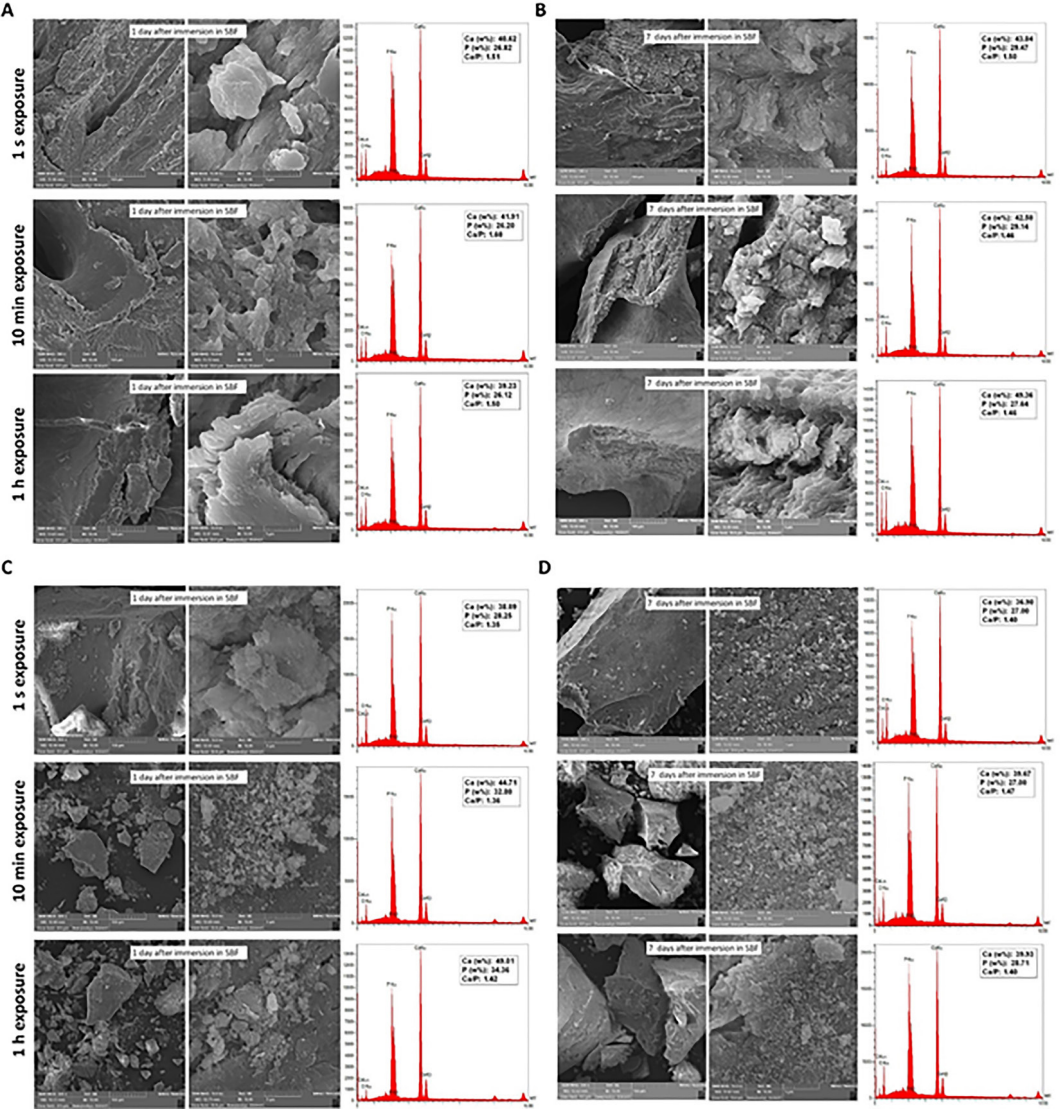
Bioactivity evaluation (SEM/EDX) of 7-day uncapping/recapping (1 s, 10 min, and 1 h in the operating room; n = 3) of the allograft (A and B) and xenograft (C and D) samples, one (A and C) and seven (B and D) days after immersion in SBF at 37 °C

 As seen in Figures 5-A and 5-B, the allograft samples exposed to the operating room air for 1 second, 10 minutes, and 1 hour and further immersed in SBF (after seven days of uncapping/recapping) for one day (Figure 5A) showed Ca/P ratios of 1.51, 1.60, and 1.50, respectively. While after seven days of immersion (Figure 5B), the values were 1.50, 1.46, and 1.46, respectively. As seen in Figures 5C and 5D, the xenograft samples exposed to operating room air for 1 second, 10 minutes, and 1 hour and further immersed in SBF (after seven days of uncapping/recapping) for one day (Figure 5C) showed Ca/P ratios of 1.35, 1.36, and 1.42, respectively. While after seven days of immersion (Figure 5B), the values were 1.40, 1.47, and 1.40, respectively. After one and seven days of immersion in SBF, no significant difference was observed in Ca/P ratios between subgroups of each bone graft with 1 s, 10 min, and 1 hour of air exposure (*P* > 0.05). Moreover, by comparing each of the variables, no significant difference was observed between the two bone graft groups. Table 1 shows Ca/P ratios one and seven days after immersing bone graft samples in SBF.

**Table 1:** Ca/P ratio after 1 and 7 days of immersing bone graft samples in SBF

**Type of graft**	**Terms of assessment**	**Exposure time to air**	**Ca/P ratio**
Allograft	1-day immersion in SBF	1 second	1.51
		10 minutes	1.60
		1 hour	1.50
	7-day immersion in SBF	1 second	1.50
		10 minutes	1.46
		1 hour	1.46
Xenograft	1-day immersion in SBF	1 second	1.35
		10 minutes	1.36
		1 hour	1.42
	7-day immersion in SBF	1 second	1.40
		10 minutes	1.47
		1 hour	1.40

## Discussion

 Operating rooms are special units that require the least possible number of microorganisms in the air. Nevertheless, these places can never be sterilized due to independent risk factors, including the type of surgery, procedure site, and staff number.^19^ Moreover, dental instruments like dental turbines and ultrasonic scalers can be considered the main sources of splatters and aerosols found in operating rooms. The splatters produced during dental procedures can increase the risk of occupational infection and contaminate dental implants and biomaterials. This contamination can cause failures in replacing the missing teeth and impose an extra charge for patients and disinfection methodologies.^20-22^ Hence, the present study aimed to evaluate two types of bone grafts from structural and microbial aspects after exposure to operating rooms for a pre-defined time.

 Calcium, phosphorus, sodium, and aluminum are inorganic contaminants, while hydrocarbons, carboxylate, and bacteria are organic. Even without direct contact with biomaterials and only through air, these contaminants can change the surface energy, chemical components, and thickness and composition of the oxide layer.^20^

 In the present study, microbiological results, including turbidity and colony formation, did not show a significant difference between the negative control group and test groups. One study demonstrated low mean microbial colony counts in samples from different operating rooms. It was also suggested that the frequency of air exchange should be augmented during complex surgeries with the increased number of personnel and indoor staff.^19^

 In the present study, bone graft vials were unpacked and uncapped/recapped in an operating room where a surgical handpiece was used at 1000 rpm. This handpiece has a much lower speed than the dental turbine, leading to fewer aerosols and infectious droplet spread. In one study, surveillance was conducted around the dental chair unit to assess the distribution or dissemination. Contamination was observed from frequent hand contact surfaces, including light handles, panel switches, syringes, and syringe holders. Furthermore, blood was detected on these surfaces though no visible signs of blood were seen.^21^ These findings make disinfection procedures even more crucial than before.

 Another study indicated that aerosol generation mainly depends on the instrument and how it is used. For instance, ultrasonic scaler aerosols can be controlled more effectively than those from a high-speed turbine.^22^ Although these aerosols and splatters can cause cross-infection and contamination, several approaches, including high-volume suction, preoperative hypochlorite use, and rubber dam isolation, were suggested to reduce aerosol generation.^23^ However, our study showed that using surgical handpieces for implant surgery did not significantly affect microbiological contamination of bone grafts. Since the humidity level of air affects the possibility and rate of particles’ displacement, the dryness of bone grafts might be one of the reasons for the absence of infection transmission from the air to bone grafts.

 Another study demonstrated that other undeniable factors, including bone grafts’ specific surface or porosity, should be considered in addition to the operation room’s conditions and surgery properties. These variables have been shown to greatly affect cell adhesion and biofilm formation.^24^

 Another objective of the present study was to assess the bioactivity changes of two types of bone grafts following air exposure. The more the bone graft’s composition is similar to human bone, the more the newly formed bone is similar to native bone. Ideally, the Ca/P ratio is 1.67. In this study, after one and seven days of immersing bone graft samples in SBF, no significant difference in Ca/P ratio was found between allograft and xenograft samples. Considering a slight increase in the Ca/P ratio of xenografts following one day of immersion in SBF, this increase can probably be attributed to the formation of hydroxyapatite crystals.

 The bioactive characteristics of bone grafts depend on calcium phosphate, Ca/P ratio, crystal structure, and solubility. Therefore, several applications, including coating technique, bone cement, and composite scaffolds, have been used to improve the bioactive features of calcium phosphate added to biomaterials.^25^ In the present study, none of the samples in both allograft and xenograft groups achieved a Ca/P ratio of 1.67. However, allografts’ Ca/P ratios were closer to this ideal value.

 In one study, different xenografts showed various amounts of Ca/P ratio. The highest (2.31) and lowest (1.22) ratios belonged to bovine-sourced xenografts.^1^ Following exposure of bone grafts to air, our findings showed no changes in the Ca/P ratios of the samples. Even minor amounts of change can be acceptable in clinical use. Hence, these changes would not play a key role in the results of biomaterial application.

## Conclusion

 Based on the results of the present study, uncapping bone graft vials and exposing them in an operating room during implant surgery did not significantly impact microbial contamination or bioactivity. Still, several conditions, such as the type of dental procedure, hand contamination during bone graft vial opening, and aerosol generation from dental instruments and surface disinfection, can greatly influence the results. Hence, the results of this study do not justify the reuse of residual grafts for the next surgeries nor recommend it. Considering the limitations of this study, future investigations assessing other crucial factors in different operating rooms and with various dental procedures are required. Moreover, disinfection and air exchange must continue to be observed during biomaterial application, bone grafting, and other routine dental treatments.

## Acknowledgments

 None.

## Availability of Data

 None.

## Competing Interests

 The authors declare that they have no competing interests.

## Ethical Approval

 The detailed study design was analyzed and approved by the Ethics Committee of the Dental School, Shahid Beheshti University of Medical Sciences, with the following ID: IR.SBMU.DRC.REC.1398.208.

## Funding

 The authors did not receive support from any organization for the submitted work.
